# Outcomes of Prostate Artery Embolisation in Patients with Severe Symptoms of Benign Prostate Hyperplasia

**DOI:** 10.21315/mjms2021.28.6.6

**Published:** 2021-12-22

**Authors:** Rohana ABDUL RAHIM, Goh ENG HONG, Nik Azuan NIK ISMAIL, Rozman ZAKARIA

**Affiliations:** 1 Department of Radiology, Faculty of Medicine, Universiti Kebangsaan Malaysia Medical Center, Cheras, Kuala Lumpur, Malaysia; 2 Department of Surgery, Faculty of Medicine, Universiti Kebangsaan Malaysia Medical Center, Cheras, Kuala Lumpur, Malaysia

**Keywords:** prostate artery embolisation, benign prostatic hyperplasia, International Prostate Symptoms Score, lower urinary tract symptoms, transurethral resection of the prostate

## Abstract

**Background:**

Benign prostatic hyperplasia (BPH) is associated with severe lower urinary tract symptoms (LUTS). The severity of LUTS is assessed by the International Prostate Symptoms Score (IPSS). Prostate artery embolisation (PAE) is a newly available method for combating LUTS. This study aims to assess the outcomes of PAE in reducing LUTS and prostate volume in patients with BPH.

**Methods:**

Patients diagnosed with BPH with severe LUTS who had undergone PAE were included. Their IPSS score was ≥ 20 despite medical therapy. PAE was performed via the unilateral femoral artery using various types of embolic material. Bilateral or unilateral embolisation of the prostate artery was considered a technical success. The severity of LUTS pre- and post-PAE were assessed using IPSS while prostate volume pre- and post-PAE were assessed by ultrasound and magnetic resonance imaging (MRI).

**Results:**

Ninety percent of patients had technical success and one required transurethral resection of the prostate (TURP). The mean IPSS reduction at the final follow-up was 12.9 (*P* < 0.028). The mean reduction of prostate volume at the last follow-up by ultrasound was 114.99 mL (*P* < 0.028) and by MRI was 29.17 mL (*P* < 0.028).

**Conclusion:**

PAE is safe and effective in reducing severe LUTS and prostate gland volume in BPH patients.

## Introduction

Benign prostatic hyperplasia (BPH) occurs in men as young as 40 years old and its growth corresponds with age. It is associated with lower urinary tract symptoms (LUTS), which include urgency, nocturia, weak stream of urine, strangury and acute urinary retention. In Malaysia, 56% of men with BPH have severe symptoms when assessed by the International Prostate Symptoms Score (IPSS) questionnaire (1). A BPH symptom score of 0–7 on the IPSS is classified as mild, score of 8–19 is classified as moderate and score of 20–35 is classified as severe (35 is the maximum score). Patients with mild symptoms are generally treated with lifestyle modification. Patients with moderate symptoms are treated with medications, namely, α-blockers and 5α-reductase inhibitors.

The non-responders, patients who cannot tolerate these drugs or patients who develop complications of BPH while receiving medical therapy, are considered for surgical therapy (2). Open surgery or transurethral resection of the prostate (TURP) is still the gold-standard surgical treatment (3). Considerations before surgery include anaesthesia risk and the complications that may arise from the surgery. The acute complications include blood loss, infection, post-operative pain and a long hospital stay of approximately 5 to 7 days, while chronic complications include strictures, sexual dysfunction, urinary incontinence or retention, short duration of symptomatic benefit and reoperation (3).

As an alternative, prostate artery embolisation (PAE) is a non-surgical way of treating BPH by blocking off the arteries that feed the gland, resulting in gland infarction and thereby reducing its size. The process is performed by an interventional radiologist. A few studies had shown that PAE is effective in reducing LUTS for patients with BPH. A study done by Pisco et al. (3) showed that PAE is a feasible procedure with short-term follow-ups demonstrating good LUTS reduction and prostate volume reduction. However, one patient developed a complication, urinary bladder wall ischaemia, and was treated surgically. Another study by Qiang et al. (4) also showed that PAE to be effective in reducing LUTS and prostate volume without any major adverse events.

As far as we know, there is no report on the effectiveness of PAE among the Malaysian population. Hence, this study was designed to assess the outcomes of PAE in reducing the LUTS in BPH patients with severe IPSS scores in one of the teaching hospitals in Kuala Lumpur, Malaysia.

## Methods

A case-series study was undertaken from February 2014 to August 2017. Ten patients with BPH with severe LUTS who fulfilled the criteria were enrolled in this study. They were referred from the urology team for having an IPSS score ≥ 20 despite medical treatment. The duration of patients on oral medication ranged from 2 weeks to 48 weeks (median duration of 15 weeks).

One of the patients had an IPSS score of 16 (moderate symptoms) but was included in this study because they needed continuous urinary bladder catheterisation due to acute urinary retention secondary to BPH, which was indicated for surgery, but PAE was chosen. All the patients were allowed to choose freely between PAE and surgical therapy. They were clearly informed of the experimental nature of the procedure. Study criteria included male BPH patient with severe LUTS (IPSS score ≥ 20) and indicated for surgery but having agreed to PAE.

The IPSS questionnaire to assess the presence, type and severity of symptoms pre- and post-PAE was given to the patient to be completed independently. Each question concerning LUTS allowed the patient to choose one of six answers indicating increasing severity of the particular symptom. The answers were assigned points from 0 to 5. Possible total scores thus ranged from 0 to 35 (asymptomatic to very symptomatic).

A mild symptom score was less than or equal to 7, while a moderate symptom score ranged from 8 to 19. A severe symptom score was any score between 20 and 35. A single question was used to assess quality of life (the answers to this question ranged from ‘delighted’ to ‘terrible’, scoring from 0 to 6). The technique, materials used and the cost of the procedure were explained to the patients.

A preliminary computed tomography angiogram of the pelvis was performed for identification, assessment of course and assessment of atherosclerotic changes of the prostate artery. Pre-PAE prostate volume was measured using ultrasound and magnetic resonance imaging (MRI). Transabdominal ultrasound was used to measure prostate volume using the formula transverse diameter (cm) × width (cm) × length (cm) × 0.52; the prostate volume by MRI was measured using the same formula. The prostate volume was measured at the longest dimension for anteroposterior (AP) diameter and longest diameter for width on the axial T2 weighted image (T2WI) and the length of the prostate was measured at the longest dimension on the T2WI sagittal view.

Similarly, for the prostate volume measurement using ultrasound, the longest dimension for AP diameter and widest diameter were taken from the axial view and the longest diameter for its length was taken on the sagittal view. The prostate gland volume measurements were performed pre-procedure and at the 3-month follow-up for both ultrasound and MRI. Toshiba and Philips ultrasound machines were used. For the MRI, a Siemens MAGNETOM Verio 3.0T was used in this study, with the sequences including T2WI sagittal, T2WI coronal, T2WI axial, T2-space coronal (3D), T1WI axial, T1WI axial contrast enhanced (CE) and Apparent diffusion coefficient (ADC)/Diffusion weighted imaging (DWI) (total time about 30 min).

The prostate volume by ultrasound and MRI were measured by a radiology resident. PAE was performed by an experienced interventional radiologist in the angiography suite. Patients were admitted 1 day before the procedure, with blood investigation taken including full blood count, renal profile and coagulation profile. The blood results were all optimised. The previous oral medications for BPH were stopped after the PAE procedure had been performed. In all patients, PAE was performed under local anaesthesia and the route of the intervention was from the right common femoral artery. Preliminary pelvic artery angiogram was performed for iliac artery and prostate artery assessment. A prophylactic single dose of ciprofloxacin was given before the procedure and continued for 1 week after PAE. The material used for the pelvic artery angiogram included an 18G puncture needle, a 5-F sheath, a 5-F Cobra-1 catheter and a 5-F SIM-1 catheter, used with a 0.035” glide wire.

Selective catheterisation of the prostatic branch of the inferior vesical arteries was performed with a phantom guidewire and a 2.7-F Progreat microcatheter. Selective angiogram of the prostatic artery was performed to a confirmed position, followed by embolisation. The embolic materials that were used included non-spherical polyvinyl alcohol (PVA) particles (Bearing and Cook, size range 4 μm–150 μm, 200 μm, 150 μm–250 μm, 250 μm–355 μm), Embozene (100 μm) and coils (Boston Contour, size range 2 mm × 2 cm, 2 mm × 3 cm, 3 mm × 3 cm, 4 mm × 2 cm and 4 mm × 4 cm). The choice of embolic material depended on the financial capability of the patients and the discretion of the interventional radiologist. The terminal finding before stopping embolisation was slow flow or near-stasis in the prostatic vessels with lesser prostatic gland opacification.

Technical success was defined as achievement of selective prostatic arterial catheterisation and embolisation on at least one side of the pelvis. For post-procedure pain relief, a regular dose of an oral non-steroidal anti-inflammatory drug (NSAID) was given for 1 day (Diclofenac sodium 50 mg). Complications were considered when they were related to the procedure (puncture site, contrast agent, sexual dysfunction and non-prostatic embolisation). Post-PAE procedure IPSS follow-ups were done at 1 month, 3 months and 2 years–2½ years. The prostate volume measurements were taken at the 3-month follow-up. The data were analysed using SPSS version 23. The Wilcoxon signed rank test was used to compare the median values between the two-time intervals, with a *P*-value of < 0.05 considered to indicate statistically significant difference.

## Results

Between the designated times of the study, 10 selected patients were enrolled, with PAE being technically successful in 9 (90%). One of the patients had a tortuous bilateral prostate artery with atherosclerotic changes resulting in difficulty in selective cannulation of the prostate artery. A second attempt was performed on a different date but a similar outcome was obtained; consequently, the procedure was abandoned and the patient underwent TURP.

The age of the patients ranged between 66 years old and 79 years old of age, with a median age of 70.5 years old. Of the nine patients, three patients were Malay (33.33%) and six patients were Chinese (66.67%). Three (33.33%) of the nine patients had unilateral PAE and six (66.67%) had bilateral PAE. Four (44.45%) of the patients were treated using a non-spherical PVA particle and coils, three (33.33%) were treated using a non-spherical PVA particle only and two (22.22%) patients were treated using Embozene with coils. None of the patients developed acute complications.

Follow-up data were available for the nine patients for both 1 month and 3 months post-PAE. Follow-ups for 2 years–2½ years were possible for six patients. The median follow-up was 25 months (range 3 months–35 months). One of the patients passed away before the 2-year follow-up due to nasopharyngeal carcinoma. Two more patients were non-contactable for the 2–2½-year follow-up.

The mean IPSS before the PAE was 23.5 ± 3.75, with a median (interquartile range [IQR]) score of 24.0 (5.75). One month after the PAE, the mean IPSS was 10.67 ± 5.00, with a median (IQR) score of 10.00 (3.00). There was a 13.50 reduction in the score (*P* < 0.008), with a percentage of reduction of 38.57%. In the IPSS results 3 months after the PAE, the mean IPSS was 7.56 ± 3.61, with a median (IQR) IPSS of 6.00 (5.00). There was, thus, a 15.94 reduction of the score (*P* < 0.08). There was also a 67.82% reduction in symptoms. For the results of IPSS 2 years–2½ years (median 25 months) after the PAE, the mean IPSS was 8.33 ± 6.02) and the median (IQR) score was 5.00 (8.75), meaning a 15.17 reduction from the pre-PAE score (*P* < 0.028). There was a 64.55% reduction in symptoms.

The mean prostate volume by ultrasound before the procedure was 116.43cm^3^ ± 103.95, with a median volume of 64.85 (133.47) cm^3^. After 3 months of PAE, the mean prostate volume by ultrasound was 58.37 cm^3^ ± 47.02, with a median volume of 47.53 (66.78) cm^3^, meaning a 49.9 % reduction in prostate volume (*P* < 0.028).

The mean prostate volume by MRI before the procedure was 88.96 ± 79.39 cm^3^, with a median (IQR) volume of 60.18 (70.36) cm^3^. Three months after PAE, the mean prostate volume by MRI was 76.47cm^3^ ± 62.72 with a median (IQR) volume of 44.02 (66.52) cm^3^, meaning a reduction in prostate volume by 14.5 cm^3^ (*P* < 0.028). There was, thus, a reduction in prostate volume of 16.07%.

**Figure 1:**
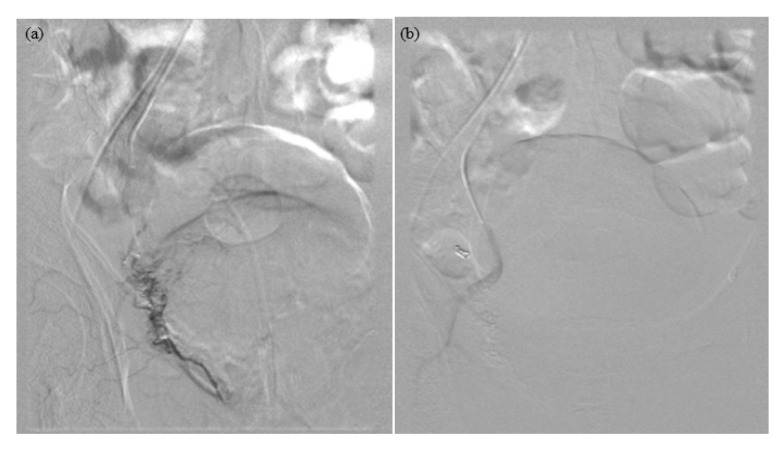
(a) Pre-PAE right prostate artery (b) Post-PAE right prostate artery

**Figure 2:**
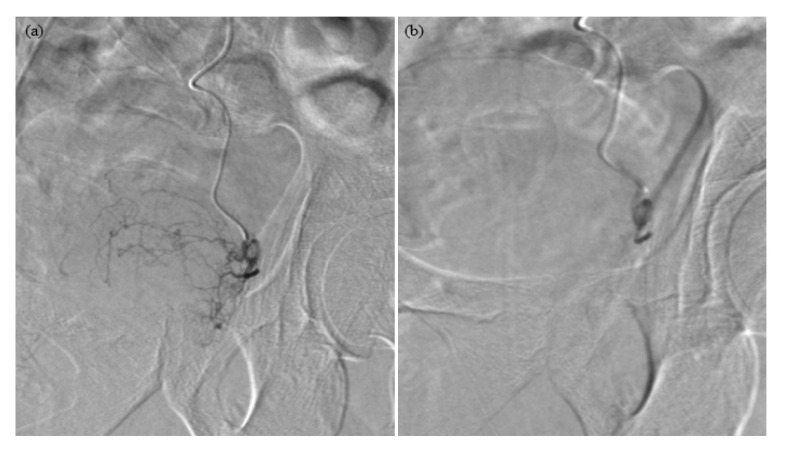
(a) Pre-PAE left prostate artery (b) Pre-PAE left prostate artery

The results for each patient are shown in Tables 1 and 2. The digital subtraction angiography images of a successful PAE are shown in Figures 1 and 2. The data are not sufficiently normally distributed due to the small number of samples. Consequently, a Wilcoxon signed rank test was used for the statistical analysis; all *P*-values were less than 0.05. This indicates a significant statistical difference for the symptoms (IPSS) and prostate volume before and after the PAE at follow-ups of 1 month, 3 months and 2 years–2½ years. During the last follow-up, two patients needed to be restarted on medical treatment. There was no need for prostatic surgery post-PAE.

## Discussion

TURP has become a standard of care for patients with severe LUTS despite maximum medical therapy. This study and data from other studies demonstrate that PAE is an effective and safe procedure for reducing severe LUTS and the volume of the prostate gland in patients with BPH (5–7).

Pisco et al. (3) reported that 14 of their 15 patients with severe BPH symptoms had a statistically significant reduction of symptoms post-PAE. In their study, one patient had a complication, bladder wall ischaemia. In another study, Bagla et al. (8) showed that PAE is effective for LUTS reduction for patients with moderate and severe symptoms of BPH. In our study, 10 patients were enrolled and nine procedures were successful. One of the patients had a failed procedure due to tortuous and atherosclerotic changes to bilateral prostate arteries. No major complications were noted.

In this study, all patients showed a reduction of the symptoms in the 1-month follow-up, with a mean IPSS reduction of 12.83 (pre-IPSS = 23.50, post-IPSS = 10.67). However, one of the patients scored only a 1 point reduction in symptoms, from 24 at pre-PAE IPSS to 23 at 1 month post-PAE; the subsequent follow-up at 3 months showed a significant reduction, from 23 to 10. However, no data were available for the 2-year follow-up. The very minimal reduction of symptoms at the 1-month follow-up likely related to the unilateral embolisation of the prostate artery, on the right side only. The left prostate artery was very tortuous, with an area of stenosis causing difficult cannulation; consequently, embolisation was abandoned. It may also have been possible that the embolic material also had a role in determining the outcome of the procedure. The non-spherical polyvinyl alcohol of size 45 μm–150 μm was used for this patient. According to the study by Hwang et al. (9), the microsphere group showed greater improvement in IPSS than did patients in the non-spherical PVA particle group. The volume of the prostate gland by ultrasound for this patient showed a minimal reduction at the 3-month follow-up, from 43.67 cm^3^ before the procedure to 40.07 cm^3^. The microsphere gives a better reduction in prostate volume than the non-spherical PVA group (9).

The overall result at 2 years–2½ years showed a significant reduction in the IPSS score, with a mean score of 8.33 (SD = 6.03), compared to the pre-PAE IPSS of 23.5 (SD = 3.75). The mean reduction IPSS compared to the pre-PAE was 15.17. When comparing the IPSS at the 3-month follow-up with the follow-up at 2 years–2½ years, three (50%) out of six patients showed increasing IPSS. However, when compared to the mean IPSS before the PAE, there is still a mean IPSS reduction of 15.17 at 2 years–2½ years. Two of the patients with increasing IPSS at 2 years–2½ years were placed on oral medication. The embolic material used for these three patients were Embozene and coils for two patients and non-spherical PVA 250 μm–350 μm for one patient. All three patients had bilateral prostatic artery embolisation.

Two of the three patients had moderate symptoms with increments in their scores of 2 and 9 (total scores of 17 and 15 at 2 years–2½ years) and one patient showed mild symptoms with an incremental score of 2 (total score of 6). However, the quality of life of these three patients remains stable. There was a significant reduction in prostate volume noted in both ultrasound and MRI follow-ups at 3 months. No repeat imaging was done at 2 years–2½ years. Other studies also showed an apparent reduction of prostate volume and stable quality of life among their populations (10, 11).

Oral NSAIDs were enough to control the pain post-procedure. In the study population, one patient with a baseline IPSS of 16 and on continuous bladder drainage (CBD) due to acute urinary retention managed to do without CBD post-PAE and the mean IPSS at the last follow-up was 8.

## Study Limitations

There are a few limitations to the present study. Not all patients were available at 2 years–2½ years because one of the patients had passed away due to other diseases and some were not contactable. Another limitation concerns the type of embolic material; the lack of standardisation may have affected the outcome. A further study should be done to assess the effectiveness of different embolic materials. A follow-up point beyond 2½ years is needed to determine the effectiveness of this procedure in the long run, and the number of subjects should be increased in order to obtain good power of study.

## Conclusion

In summary, PAE for BPH patients is an effective and safe procedure for combating severe LUTS. In this study, patients with unilateral and bilateral prostate artery embolisation showed a significant reduction in symptoms and size of the prostate gland.

## Figures and Tables

**Table 1:** Follow-ups profile for patients enrolled in the study

No. of patient	Volume (USG)		Volume (MRI)		IPSS				Successful PAE	Embolic material

	Pre-PAE	Post-PAE 3 months	Pre-PAE	Post-PAE 3 months	Pre-PAE	Post-PAE 1 month	Post-PAE 3 months	Post-PAE 2–2.5 year	Successful PAE	Embolic material
1	84.88	54.36	72.75	41.33	27	10	6	4	Bilateral	PVA-Bearing 150 μm–250 μm–> R, PVA-Cook 200 μm–> L
2	-	-	47.6	46.70	24	11	15	17 Restart on medication	Bilateral	PVA-Bearing 250 μm–350 μm
3	43.64	40.7	17.07	11.4 (at 1 month)	24	23	10	-	Unilateral	PVA-Bearing 45 μm–150 μm
4	200.93	99.18	-	82.00	26	7	4	15 Restart on medication	Bilateral	Embozene 100 μm and coil 4 mm × 2 cm–> R, Embozene 100 μm and coil 4 mm × 4 cm–> L
5	318.99	129.16	235.79	188.22	21	9	4	6	Bilateral	Embozene 100 μm and coil
6	168.31	-	185.08	131.34	28	6	5	4	Bilateral	PVA-Bearing 150 μm–250 μm, coil 2 mm × 3 cm
7	44.82	-	84.72	-	21	10	6	Passed away	Unilateral	PVA 150 μm–250 μm, coil 2 mm × 3 cm
8	28.82	12.1	24.16	12.9	21	12	10	1	Bilateral	PVA Boston Contour 250 μm–355 μm, coil 2 mm × 3 cm
9	41.05	14.7	44.55	32.79	16	8	8	-	Unilateral	PVA-Bearing 150 μm–250 μm, coil 3 mm × 3 cm
10	-	-	-	-	27	-	-	-	Abandoned due to tortuous and severe arteriosclerotic changes of bilateral prostate arteries	

**Table 2:** The results for the IPSS and prostate volumes post-PAE follow-ups

Variables	*N*	Mean	SD	Median	IQR	Minimum	Maximum	Wilcoxon signed rank test	*P*-value
IPSS									
Pre-PAE	10	23.50	3.75	24.00	5.75	16	28		
Post-PAE (1 month)	9	10.67	5.00	10.00	3.00	6	23	−2.666	0.008
Post-PAE (3 months)	9	7.56	3.61	6.00	5.00	4	15	−2.666	0.008
Post-PAE (2–2½ years)	6	8.33	6.02	5.00	8.75	4	17	−2.201	0.028
Prostate volume USG (mL)									
Pre-PAE	8	116.43	103.95	64.85	133.47	28.82	318.99		
Post-PAE (3 months)	6	58.37	47.02	47.53	66.78	12.10	129.16	−2.201	0.028
Prostate volume MRI (mL)									
Pre-PAE	8	88.97	79.40	60.18	70.36	17.07	235.79		
Post-PAE (3 months)	7	76.47	62.72	44.02	66.52	12.90	188.22	−2.201	0.028

*Notes:* Vol = volume; USG = ultrasonography; MRI = magnetic resonance imaging
